# Carbonic anhydrase inhibitor attenuates ischemia-reperfusion induced acute lung injury

**DOI:** 10.1371/journal.pone.0179822

**Published:** 2017-06-23

**Authors:** Chou-Chin Lan, Chung-Kan Peng, Shih-En Tang, Kun-Lun Huang, Chin-Pyng Wu

**Affiliations:** 1 Division of Pulmonary Medicine, Department of Internal Medicine,Taipei Tzu Chi Hospital, Buddhist Tzu Chi Medical Foundation, New Taipei City, Taiwan, Republic of China; 2 School of Medicine, Tzu-Chi University, Hualien, Taiwan, Republic of China; 3 Division of Pulmonary Medicine, Department of Internal Medicine,Tri-Service General Hospital, Taipei, Taiwan, Republic of China; 4 Institute of Undersea and Hyperbaric Medicine, National Defense Medical Center, Taipei, Taiwan, Republic of China; 5 Department of Critical Care Medicine, Li-Shin Hospital, Tao-Yuan County, Taiwan, Republic of China; Hospital for Sick Children, CANADA

## Abstract

Ischemia-reperfusion (IR)-induced acute lung injury (ALI) is implicated in several clinical conditions including lung transplantation, cardiopulmonary bypass surgery, re-expansion of collapsed lung from pneumothorax or pleural effusion and etc. IR-induced ALI remains a challenge in the current treatment. Carbonic anhydrase has important physiological function and influences on transport of CO_2_. Some investigators suggest that CO_2_ influences lung injury. Therefore, carbonic anhydrase should have the role in ALI. This study was undertaken to define the effect of a carbonic anhydrase inhibitor, acetazolamide (AZA), in IR-induced ALI, that was conducted in a rat model of isolated-perfused lung with 30 minutes of ischemia and 90 minutes of reperfusion. The animals were divided into six groups (n = 6 per group): sham, sham + AZA 200 mg/kg body weight (BW), IR, IR + AZA 100 mg/kg BW, IR + AZA 200 mg/kg BW and IR+ AZA 400 mg/kg BW. IR caused significant pulmonary micro-vascular hyper-permeability, pulmonary edema, pulmonary hypertension, neutrophilic sequestration, and an increase in the expression of pro-inflammatory cytokines. Increases in carbonic anhydrase expression and perfusate pCO_2_ levels were noted, while decreased Na-K-ATPase expression was noted after IR. Administration of 200mg/kg BW and 400mg/kg BW AZA significantly suppressed the expression of pro-inflammatory cytokines (TNF-α, IL-1, IL-6 and IL-17) and attenuated IR-induced lung injury, represented by decreases in pulmonary hyper-permeability, pulmonary edema, pulmonary hypertension and neutrophilic sequestration. AZA attenuated IR-induced lung injury, associated with decreases in carbonic anhydrase expression and pCO_2_ levels, as well as restoration of Na-K-ATPase expression.

## Introduction

Exposure of the lungs to periods of ischemia and the initiation of reperfusion causes ischemia-reperfusion (IR)-induced acute lung injury (ALI)[1]. IR-induced ALI is an important issue in lung transplantation[1], which provides a potential cure for patients with end-stage pulmonary diseases. Despite advances in organ preservation and peri-operative care, IR-induced ALI remains a significant cause of post-transplantation mortality and morbidity[2]. It has been widely accepted that effective organ preservation is one of the keys to successful lung and heart-lung transplantation[2]. Above and beyond of lung transplantation, IR-induced ALI also affects treatment of cardiopulmonary bypass and re-expansion of collapsed lung associated with pneumothorax or pleural effusion[3–5] The mortality associated with IR-induced ALI remains high despite the availability of modern treatments. Thus, there is a need for -more effective treatments for IR-induced ALI.

Carbonic anhydrase is an enzyme that assists rapid interconversion of carbon dioxide (CO_2_) and water into carbonic acid, protons and bicarbonate ions[6–8]. It is widely distributed in lung tissues including Type I and II alveolar epithelial cells, capillary endothelial cells, and red blood cells[8, 9]. In the lungs, carbonic anhydrase turns the carbonic acid into CO_2_ to be exhaled. Inhibition of carbonic anhydrase activities by acetazolamide (AZA) delaysCO_2_-mediated pHi decreases, which suggests that there is carbonic anhydrase activity in alveolar epithelial cells with influence of CO_2_ level[8].

In patients with acute respiratory failure, impairment of gas exchange may lead to accumulation of CO_2_ in the lungs. It is suggested that hypercapnia may deteriorate lung function[10], where CO_2_ can act as a signaling molecule via pH-independent mechanisms resulting in deleterious effects on epithelial and endothelial barriers, clearance of pulmonary edema, innate immunity, and host defense[10]. Since carbonic anhydrase catalyzes the interconversion between CO_2_ and bicarbonate[11], we postulated that carbonic anhydrase may have a role in IR-induced ALI.

AZA, a sulfonamide derivative, is a carbonic anhydrase inhibitor[6] that has been shown to be effective in the treatment of glaucoma by inhibiting of HCO_3_^–^ production[2]. AZA is also as a diuretics, antiepileptics, treatment for idiopathic intracranial hypertension, and mountain sickness[6, 12]. However, the therapeutic effects of AZA in IR-induced ALI are not yet known.

Therefore, by applying AZA, we conducted the isolated lung model of IR to study the therapeutic effects of carbonic anhydrase inhibitors in IR-induced ALI. We measured perfusate blood gas, expression and activity of carbonic anhydrase, expression of Na,K-ATPase, pulmonary vascular permeability, pulmonary arterial pressure (PAP), lung histopathology, pulmonary edema, total protein, and proinflammatory cytokines in these rats. Understanding the role of carbonic anhydrase in IR-induced ALI may be helpful in developing new strategies of carbonic anhydrase inhibitors in treating lung injury.

## Materials and methods

### Isolation and perfusion of lungs

The National Science Council and the Animal Review Committee of the National Defense Medical Center (NDMC), Taipei, Taiwan, approved our study protocol. Animals in this study were cared in accordance with the ‘‘Guide for the Care and Use of Laboratory Animals” published by the United States’ National Institutes of Health. Sprague-Dawley rats used for our experiment were from BioLASCO Taiwan Co., Ltd and were housed in individually ventilated cages (450*290*265 mm). Rats were housed at a temperature of 22 ± 1°C and humidity of 60 ± 10%, with standard bedding, and maintained on a 12-h light-dark cycle in the animal facilities at the NDMCLaboratory Animal Center (AAALAC accreditation).

Rats were fed a normal chow diet (Laboratory Autoclavable Rodent diet 5010 with 27.5% protein, 13.5% fat, 59% carbohydrate). This was a non-survival study that utilized deep anesthesia to isolate lung. The rats were without pain sensation and were euthanized at the end of the experiment.

Male rats were anesthetized through intra-peritoneal injection of zoletil (20 mg/kg). After deep anesthesia was confirmed, tracheostomy was performed and a cannula was inserted into the trachea. The lungs were ventilated with a humidified gas mixture containing 5% CO_2_ in air at a frequency of 60 cycle/min, a tidal volume of 8 mL/kg, and an end-expiratory pressure of 1 cm H_2_O.

**Fig 1:**
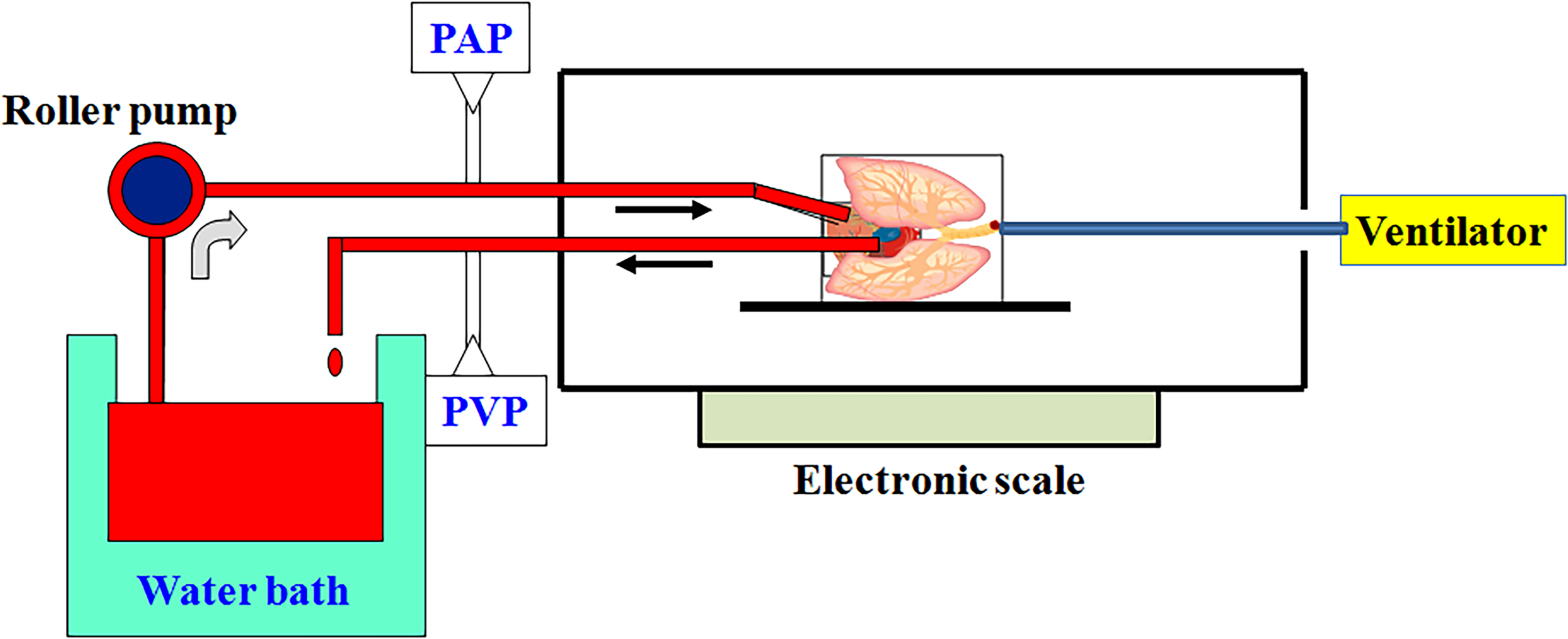
Schematic diagram of the isolated lung model. The rats were ventilated via tracheostomy using a rodent ventilator. The chest was opened by median sternotomy, and 10–12 mL of intracardiac blood was withdrawn via left atrial appendage. One catheter was inserted into the left atrium via a left ventriculotomy. This catheter was placed in the left atrium to collect effluent perfusate for re-circulation. Subsequently, a catheter was inserted into the main pulmonary artery (PA) via the right ventriculotomy. The other end of the catheter was connected to a tube for pulmonary perfusion. PA pressure (PAP) and pulmonary venous pressure (PVP) were measured using a pressure sensor, that was connected to the catheter. A peristaltic pump was used to perfuse the lungs with re-circulated perfusate. The perfusion rate was kept at 8–10 mL/min by a roller pump while constant temperature (37°C) was maintained by a water bath.

Fig 1 depicts the experimental procedure schematic for the isolation and perfusion of lungs. The chest was opened by median sternotomy, and heparin (1 U/g of body weight, BW) was injected into the right ventricle to prevent blood coagulation. 10–12 mL of intracardiac blood was withdrawn via left atrial appendage. Following a small incision on the left ventricle, a catheter was inserted into the left atrium to collect effluent perfusate for re-circulation. Subsequently, a catheter was inserted into the main pulmonary artery (PA) via the right ventriculotomy. The PA and ascending aorta were tied together with a silk suture. PAP and pulmonary venous pressure (PVP) were measured with a pressure sensor, that was connected to the catheter. The other end of the catheter was connected to a tube for pulmonary perfusion.

A peristaltic pump (Model 1203, Harvard Apparatus) was used to perfuse the lungs with re-circulated perfusate composed of blood (10–12 mL) mixed 1:1 with physiologic salt solution (119 mM NaCl, 4.7 mM KCl, 1.17 mM MgSO_4_, 22.6 mM NaHCO_3_, 1.18 mM KH_2_PO_4_, 1.6 mM CaCl_2_, 5.5 mM glucose, and 50 mM sucrose). Bovine albumin (4 g/dL) was added to maintain the osmolarity of the perfusate. Perfusion rate was kept at 8–10 mL/min using a roller pump while constant temperature of 37°C was maintained by a water bath. The preparation was placed on an electronic balance with the isolated lungs remaining *in situ*.

### Experimental protocols for IR-induced ALI

The IR-induced ALI was performed with 30 minutes of ischemia by stopping ventilation and perfusion. Following 30-minutes of ischemia, the lungs were re-perfused for 90 minutes and ventilated with 5% CO_2_ + 95% air.

The animals were divided into six groups (n = 6 per group): sham, sham + AZA200 mg/kg BW) (sham-A200), IR, IR+AZA100mg/kg BW (IR-A100), IR+AZA200mg/kg BW (IR-A200) and IR+AZA400mg/kg BW (IR-A400). AZA (Sanwa Kagaku Kenkyusho Co.,Ltd, Japan) was 500mg dissolved in 5mL normal saline according to the instruction. AZA was administered via perfusate at the initiation of reperfusion. The rats were analyzed for perfusate blood gas, micro-vascular permeability (Kf), lung histopathology, lung W/D, total protein concentration, cytokines (TNF-α, IL-1, 6, and 17), expression and activity of carbonic anhydrase and expression of Na,K-ATPase.

### Perfusate gas analysis

Perfusate blood gas was measured (Radiometer America Inc., Westlake, OH) at baseline, post-ischemia 30 minutes, post-reperfusion 90 minutes in the sham, IR and IR-A400 groups.

### Microvascular permeability (Kf)

An index of Kf was determined from the changes in lung weight(LW) induced by elevated PVP as previously described[13]. During ventilation and perfusion, the PVP was rapidly elevated by 10 cm H_2_O for 7 minutes. The slow, and steady phase of LW gain as a function of time (ΔW/ΔT) was plotted on a semi-logarithmic scale and then extrapolated to time zero to obtain the initial rate of trans-capillary filtration. From this plot, Kf was defined as the y-intercept (gm/min) divided by PVP (10 cm H_2_O) and LW (gm), and expressed in whole units of grams per minute per centimeter of H_2_O multiplied by 100 g[13].

### Total protein concentration in bronchoalveolar lavaged fluid (BALF)

At the end of the experiment, the lungs were lavaged twice with 2.5 mL of isotonic saline. The returned fluid was collected by free drainage. The BALF was centrifuged at 200 x g for 10 minutes, and total protein concentration in the supernatant was determined using bicinchoninic acid protein assay (Pierce, Rockford, IL, USA).

### Pro-inflammatory cytokine levels in perfusate

The expression levels of pro-inflammatory cytokines including TNF-α, IL-1, 6, and 17 in perfusate was measured from all rats by commercially available enzyme-linked immuno-sorbent assay (R&D Systems Inc., Minneapolis, MN). Time course of these cytokines was also measured at baseline, post-ischemia 30 minutes and post-reperfusion 90 minutes in the sham, IR and IR-A400 groups.

### Pulmonary edema

After the experiment, the right lung tissue was removed and the wet weight was determined. It was then dried in an oven at 60°C for 48 hours. The wet and dry weights were then used to calculate the lung’s W/D.

### Lung histopathology

The lungs were fixed with 10% formaldehyde at 20 cmH_2_O infused through the trachea. The tissues were immersed in 10% formaldehyde fixative for 24 hours, embedded in paraffin wax, and then cut into 4–6 μm-thick sections with a microtome. The sections were stained with hematoxylin and eosin (H&E) to assess lung inflammation and edema.

### Lung neutrophil quantification

H&E -stained lung sections were used to quantify the number of neutrophils per high power field (400X)[14]. For each slide, neutrophils were counted in 10 non-overlapping high power fields. Each slide was examined by an observer blinded to the study[15].

### mRNA expression assay for carbonic anhydrase II

We investigated the expression of carbonic anhydrase II mRNA in the lung tissues by quantitative real-time polymerase chain reaction (PCR). Carbonic anhydrase II because is the most common form of carbonic anhydrase in lung tissues[8].

Isolation of mRNA was performed using the kit (RNA purification kit: ZYMO Direct-zol^TM^ RNA MiniPrep) according to manufacturer’s instructions. The quantitative PCR amplification reaction was performed using a thermal cycler (Eco Real-Time PCR system, Illumina). Primer sequences to amplify carbonic anhydrase II were synthesized according to Rn01462065_m1. ß-actin was used as a reference gene while data were normalized using the comparative cycle-threshold method.

### Immunofluorescent staining of carbonic anhydrase II

Formalin-fixed paraffin sections of rat lung (4-μm thickness) were deparaffinized before antigen retrieval. Endogenous peroxidase was blocked by incubating in 3% of H_2_O_2_ in methanol for 15 minutes. The slides were then incubated for 60 minutes with a carbonic anhydrase II rabbit polyclonal antibody. After washing, slides were sequentially incubated with rat tissue specific horseradish peroxidase-polymer anti-rabbit antibody (Nichirei Corporation) for 30 minutes. Images were captured using a fluorescence microscope (Leica/DM 2500) equipped with an EMCCD camera, using SPOT 4.7 advanced imaging software.

### Carbonic anhydrase activity

Carbonic anhydrase activity by modified Hansson's procedure was performed as previously described[16]. Sections mounted on glass microscope slides were dipped for 2 second into incubation media containing 5.85 mmol/L KH_2_PO_4_, 157 mmol/L NaHCO_3_, 53 mmol/L H_2_SO_4_, 8.75 mmol/L CoSO_4_, and 0.5% Triton X-100, pH 5.9. After dipping, the slides were held in front of a current of moving air for 28 second. Dipping and aerating were repeated 15 times. Aeration facilitated the evolution of CO_2_ produced locally at sites of active carbonic anhydrase. With the loss of CO_2_, these sites become alkaline, causing the precipitation of CoPO_4_, which, in turn, is visualized by incubation in 0.5% (NH_4_)S.

### Membranous Na,K-ATPase in lung tissues

Lung tissues were homogenized on ice in a homogenization buffer containing sucrose (250mM), triethanolamine (10 mM), EDTA (1 mM), EGTA (5 mM), NaF (50 mM), Na_3_VO_4_ (1mM). Homogenized lung samples were sonicated on ice and centrifuged at 600 g for 3 min. The supernatant were centrifuged at 6000 g for 3 minutes. The supernatant were further centrifuged at 18000 g for 10 minutes. Cell membrane was the pellet dissolved with 100μl and was used for Western blot analysis of Na,K-ATPase α1. Equal amounts of lung homogenates (30μg/lane) were fractionated on 10% sodium dodecyl sulfate–polyacryl-amide gel electrophoresis and transferred to Hybond polyvinylidene fluoride membranes. The membranes were blocked by incubation in PBS containing 0.1% Tween 20 and 5% nonfat milk for 1 hour at room temperature. Blots were incubated with an antibodies to Na,K-ATPase α1 (Bioworld Technology) overnight at 4°C. The blots were then washed three times for 10 minuteseach in PBS containing 0.1% Tween 20. The blots were incubated with horseradish peroxidase linked anti-rabbit immunoglobulin G (1:40,000) at room temperature for one hour, and then washed three times in PBS containing 0.1% Tween 20 for 10 minutes. Bands were visualized using enhanced chemiluminescence reagents and exposure to radiography film. The blots were then stripped and incubated with an anti-TATA antibody to ensure equal loading.

### Data analysis

All statistical analyses were performed using the SPSS software 18.0 (SPSS Inc., Chicago, IL). All the values are presented as box plots with median and quartiles. Differences between groups were evaluated using Kruskal–Wallis followed by post hoc comparisons with Games Howell tests (intergroup comparison). Repeated-measures analysis of variance (ANOVA) was used to analyze differences among time course of blood gas and pro-inflammatory cytokines in perfusate. Statistical significance was set at p<0.05.

## Results

### AZA decreased pCO2 in perfusate after IR

**Fig 2:**
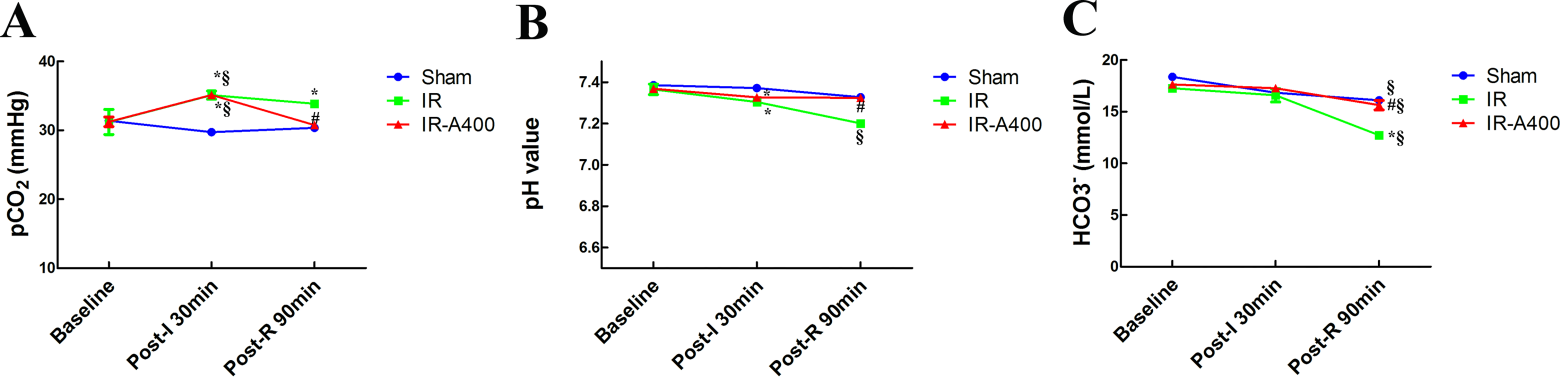
Changes of pCO_2_, pH and HCO_3_^-^ and in perfuate. The mean pCO_2_, pH, and HCO3^-^ values at baseline were similar across all rat groups. (A) In the IR and IR-A400 groups, pCO_2_ levels increased after ischemia (*p*<0.05 as compared with baseline). Following reperfusion and ventilation, pCO_2_ levels in the IR-A400 group were lower than that in the IR group (p<0.05 as compared between the IR and IR-A400 groups). (B) The pH was stationary in the sham and IR-A400 groups. However, pH decreased in the IR group (p<0.05 as compared with baseline; p<0.05 as compared between the IR and sham, and IR-A400 groups). (C) HCO_3_^-^ had similar changes as pH during ischemia and reperfusion. There was a significant difference from the *Sham (*p*<0.05) and ^#^IR (*p*<0.05) groups and from ^§^baseline (*p*<0.05). Sham group; IR, ischemia-reperfusion group; IR-A400, IR + AZA 400mg/kg BW group.

Changes of pH, pCO_2_ and HCO_3_^-^ in perfusate at baseline, post-ischemia, and post-reperfusion are shown in Fig 2. At baseline, pCO_2_, pH, and HCO_3_^-^ values were similar across all rat groups. In the IR and IR-A400 groups, pCO_2_ were increased after ischemia (*p*<0.05 as compared with baseline). Following reperfusion, pCO_2_ in the IR-A400 group was significantly lower than that in the IR group (p<0.05 as compared between the IR and IR-A400 groups). The pH was stationary in both sham and IR-A400 groups (p>0.05 as compared with baseline), but showed a significant downward trend in the IR group (p<0.05 as compared with baseline). HCO_3_^-^ levels demonstrated changes similar to those observed in pH levels during both ischemia and reperfusion in the IR group.

### AZA decreased IR-induced pulmonary edema

**Fig 3:**
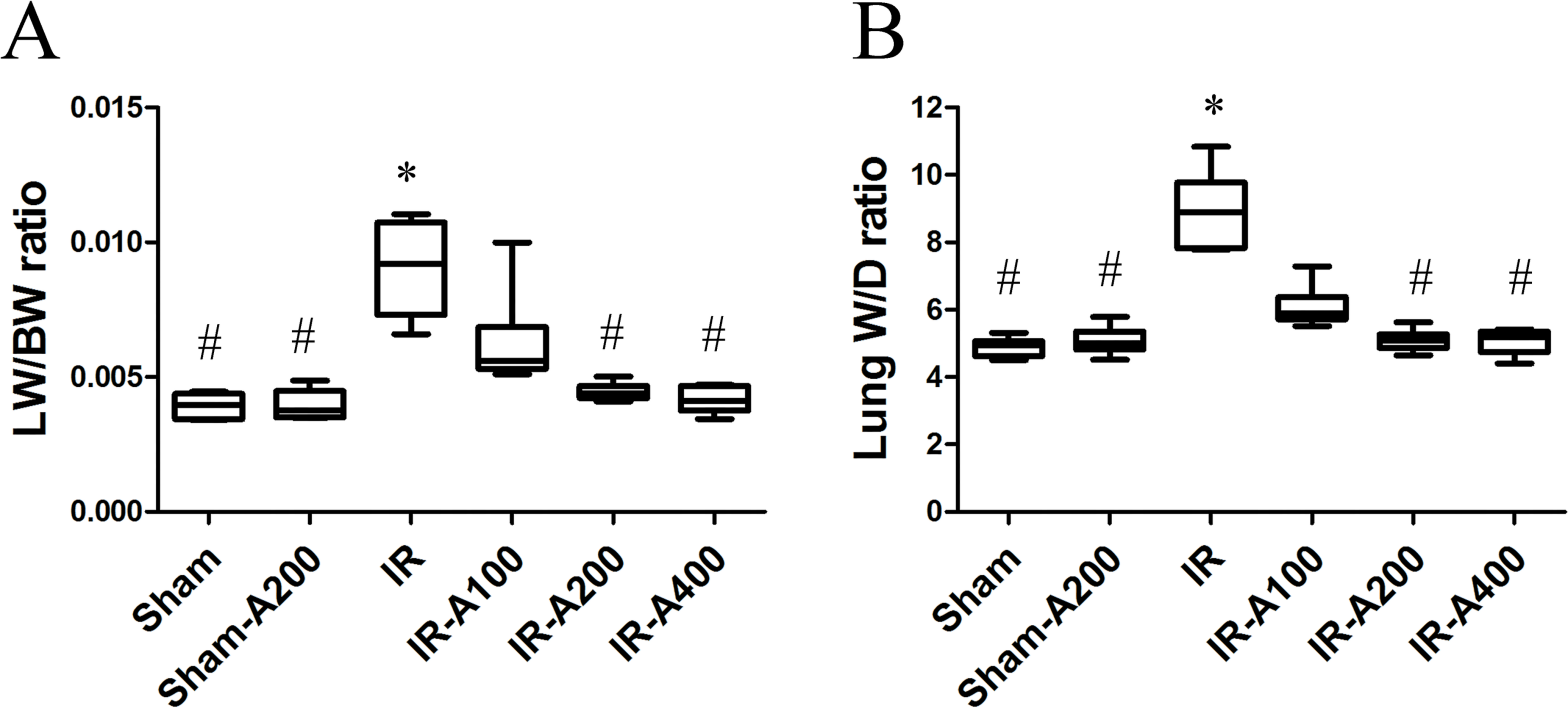
AZA decreased IR-induced pulmonary edema. Lung weight/body weight (LW/BW) (A) and wet/dry weight (W/D) (B) ratios were significantly higher in the IR than sham groups (*p*<0.05 as compared between the IR and sham). The IR-A200 and IR-A400 groups had significantly lower LW/BW and W/D than the IR group (*p*<0.05 as compared between the IR and IR with AZA groups). There was a significant difference from the * Sham (*p*<0.05) and ^#^IR (*p*<0.05) groups. Sham group; Sham-A200, Sham + AZA 200mg/kg BW group; IR, ischemia-reperfusion group; IR-A100, IR + AZA 100mg/kg BW group; IR-A200, IR + AZA 200mg/kg BW group; IR-A400, IR + AZA 400mg/kg BW group.

Pulmonary edema was assessed by lung-weight-to-body-weight (LW/BW) and W/D ratios. LW/BW (Fig 3A) and W/D ratios (Fig 3B) were significantly higher in the IR group (*p*<0.05 as compared between the sham and IR groups). AZA 200 mg/kg and 400 mg/kg significantly decreased LW/BW and W/D ratios after IR (*p*<0.05 as compared between IR and IR with AZA).

### AZA decreased IR-induced microvascular hyper-permeability

**Fig 4:**
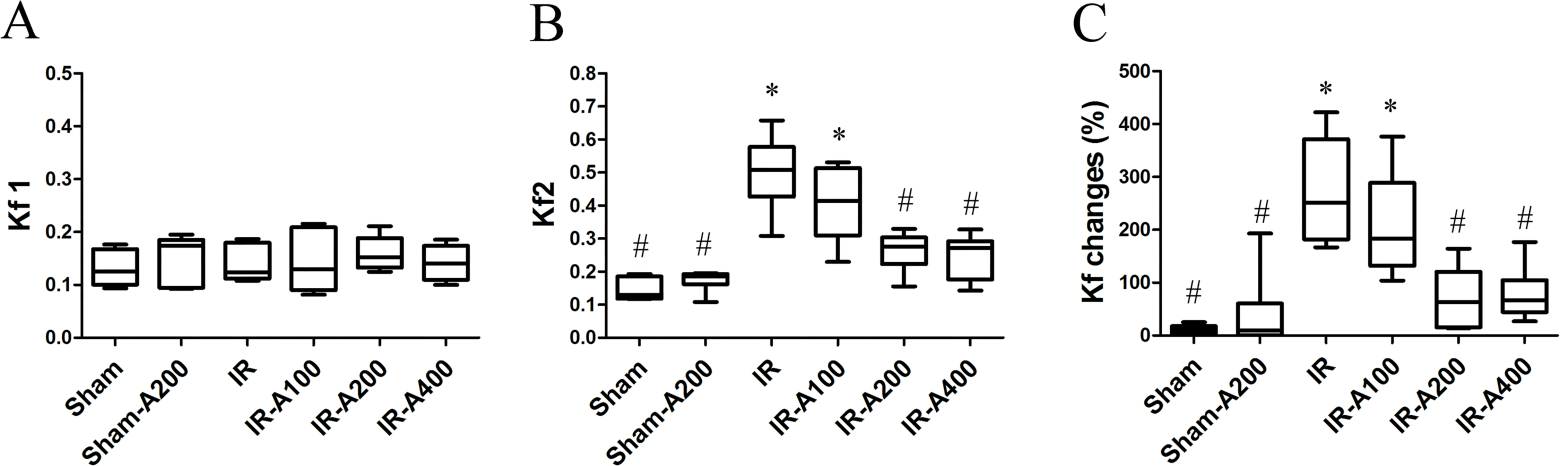
AZA decreased IR-induced microvascular hyper-permeability. The Kf1 at baseline was similar among all groups (*p*>0.05 as compared among the sham, IR and IR with AZA groups). (B) The Kf2 in the IR group was significantly higher than that in the sham group (*p*<0.05 as compared between the IR and sham groups) (Fig 2B). AZA 200mg/kg BW and 400mg/kg BW significantly attenuated IR-induced microvascular hyper-permeability (*p*<0.05 as compared with the IR group). (C) Changes of micro-vascular permeability were significantly higher in the IR group (*p*<0.05 as compared with the sham group) and were attenuated by administration of AZA 200mg/kg BW and 400 mg/kg BW (*p*<0.05 as compared between the IR and IR with AZA groups). There was a significant difference from the * Sham (*p*<0.05) and ^#^IR (*p*<0.05) groups. Sham group; Sham-A200, Sham + AZA 200mg/kg BW group; IR, ischemia-reperfusion group; IR-A100, IR + AZA 100mg/kg BW group; IR-A200, IR + AZA 200mg/kg BW group; IR-A400, IR + AZA 400mg/kg BW group.

The Kf1 at baseline was similar among all groups (*p*>0.05 as compared among the sham, IR and IR with AZA groups) (Fig 4A). The Kf2 in the IR group was significantly higher than that in the sham group (*p*<0.05 as compared with the sham group) (Fig 4B). AZA 200 mg/kg and 400 mg/kg significantly attenuated IR-induced micro-vascular hyper-permeability (*p*<0.05 as compared with the IR group). Changes of micro-vascular permeability were significantly higher in the IR group (*p*<0.05 as compared with the sham group) and were attenuated by AZA 200mg/kg BW and 400 mg/kg BW (*p*<0.05 as compared between the IR and IR with AZA)(Fig 4C).

### AZA decreased IR-induced pulmonary hypertension

**Fig 5:**
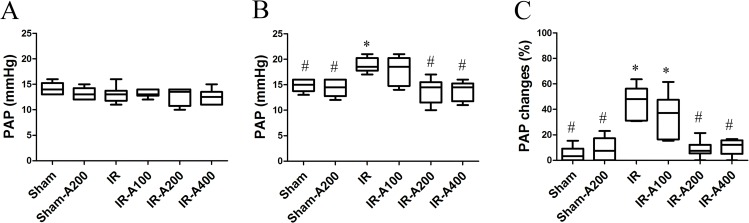
AZA decreased IR-induced pulmonary hypertension. (A) The PAP at baseline was similar among these groups (*p*>0.05 as compared among the sham, IR and IR with AZA groups). (B) After IR, the PAP levels were significantly increased in the IR group (*p*<0.05 as compared with the sham group). AZA 200mg/kg BW and 400mg/kg BW significantly decrease the PAP levels after IR (*p*<0.05 as compared between the IR and IR with AZA groups). (C) Changes of PAP levels were significantly increased in the IR group (*p*<0.05 as compared with the sham group) and were attenuated by AZA 200mg/kg BW and 400 mg/kg BW (*p*<0.05 as compared between the IR and IR with AZA groups). There was a significant difference from the * Sham (*p*<0.05) and ^#^IR (*p*<0.05) groups. Sham group; Sham-A200, Sham + AZA 200mg/kg BW group; IR, ischemia-reperfusion group; IR-A100, IR + AZA 100mg/kg BW group; IR-A200, IR + AZA 200mg/kg BW group; IR-A400, IR + AZA 400mg/kg BW group.

The PAP levels at baseline were similar among all groups (*p*>0.05 as compared among the sham, IR and IR with AZA groups)(Fig 5A). After IR, the PAP levels were significantly higher in the IR group (*p*<0.05 as compared with the sham group)(Fig 5B). AZA 200 mg/kg and 400 mg/kg significantly decreased the PAP levels after IR (*p*<0.05 as compared between the IR and IR with AZA groups). Changes of PAP levels were significantly higher in the IR group than in the sham group (*p*<0.05 as compared between the sham and the IR groups) and were attenuated by AZA 200mg/kg and 400 mg mg/kg (*p*<0.05 as compared between the IR and the IR with AZA groups) (Fig 5C).

### AZA attenuated lung injury

**Fig 6:**
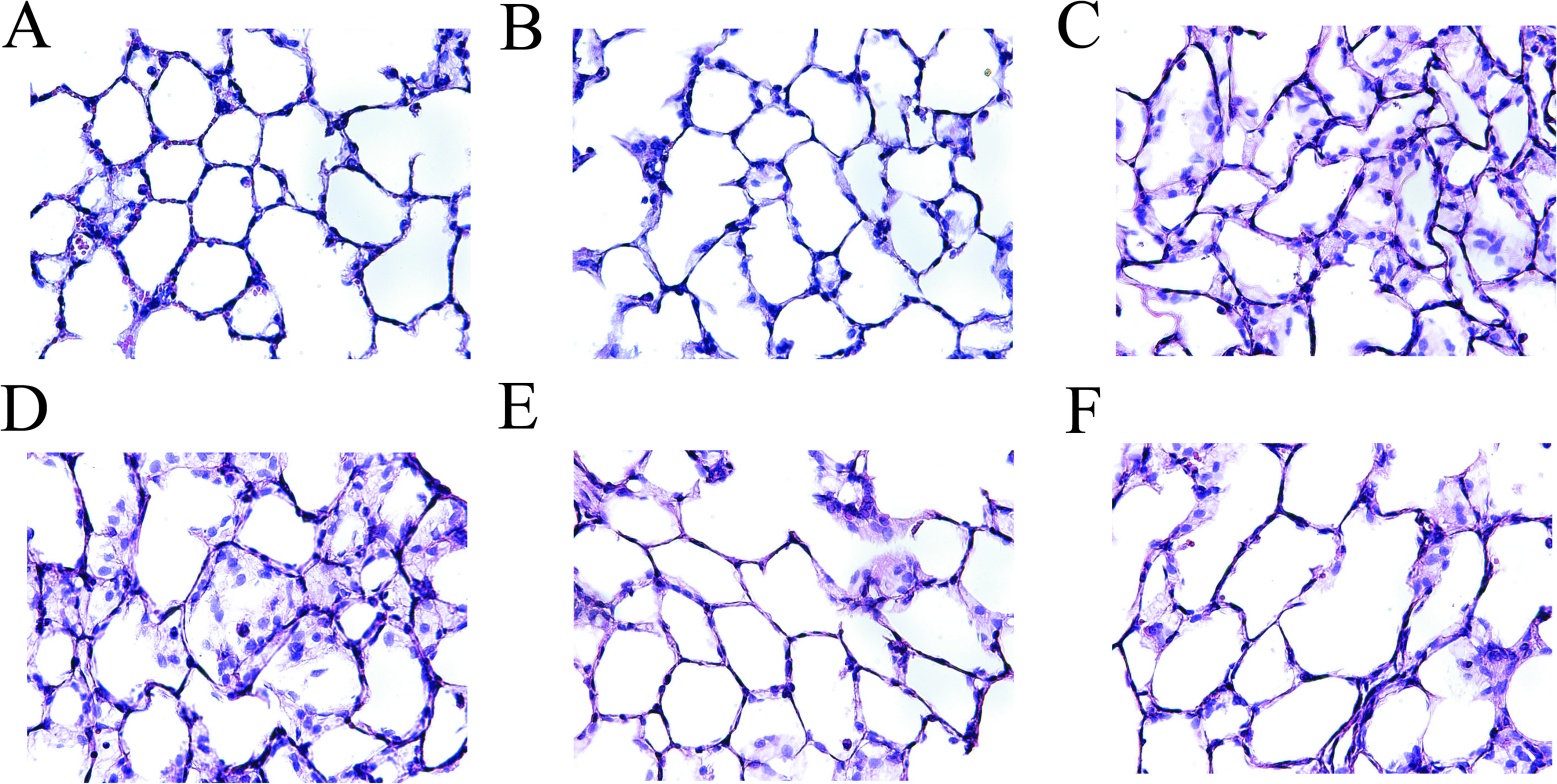
AZA attenuated lung injury. The sham (A) and sham-A200 (B) groups had normal histology while the IR (C) and IR-A100 (D) groups showed prominent neutrophilic sequestration, inter-alveolar septum thickening and pulmonary edema. IR-induced neutrophilic sequestration, inter-alveolar septum thickening and pulmonary edema were markedly attenuated by AZA 200 mg/kg BW (E) and 400 mg/kg BW (F).

The sham (Fig 6A) and sham-A200 groups (Fig 6B) demonstrated normal pulmonary histology while those in the IR (Fig 6C) and IR-A100 (Fig 6D) groups showed prominent neutrophilic sequestration, inter-alveolar septum thickening and pulmonary edema. IR-induced neutrophilic sequestration, inter-alveolar septum thickening and pulmonary edema were markedly attenuated by AZA 200 mg/kg (Fig 6E) and 400 mg/kg (Fig 6F).

### AZA attenuated neutrophilic sequestration in IR-induced ALI (Fig 7)

Neutrophilic sequestration was markedly increased in the IR group (*p*<0.05 as compared with the sham group). Neutrophilic sequestration was significantly decreased by AZA 200 mg/kg BW and 400 mg/kg BW after IR (*p*<0.05 as compared between the IR and the IR with AZA groups).

**Fig 7:**
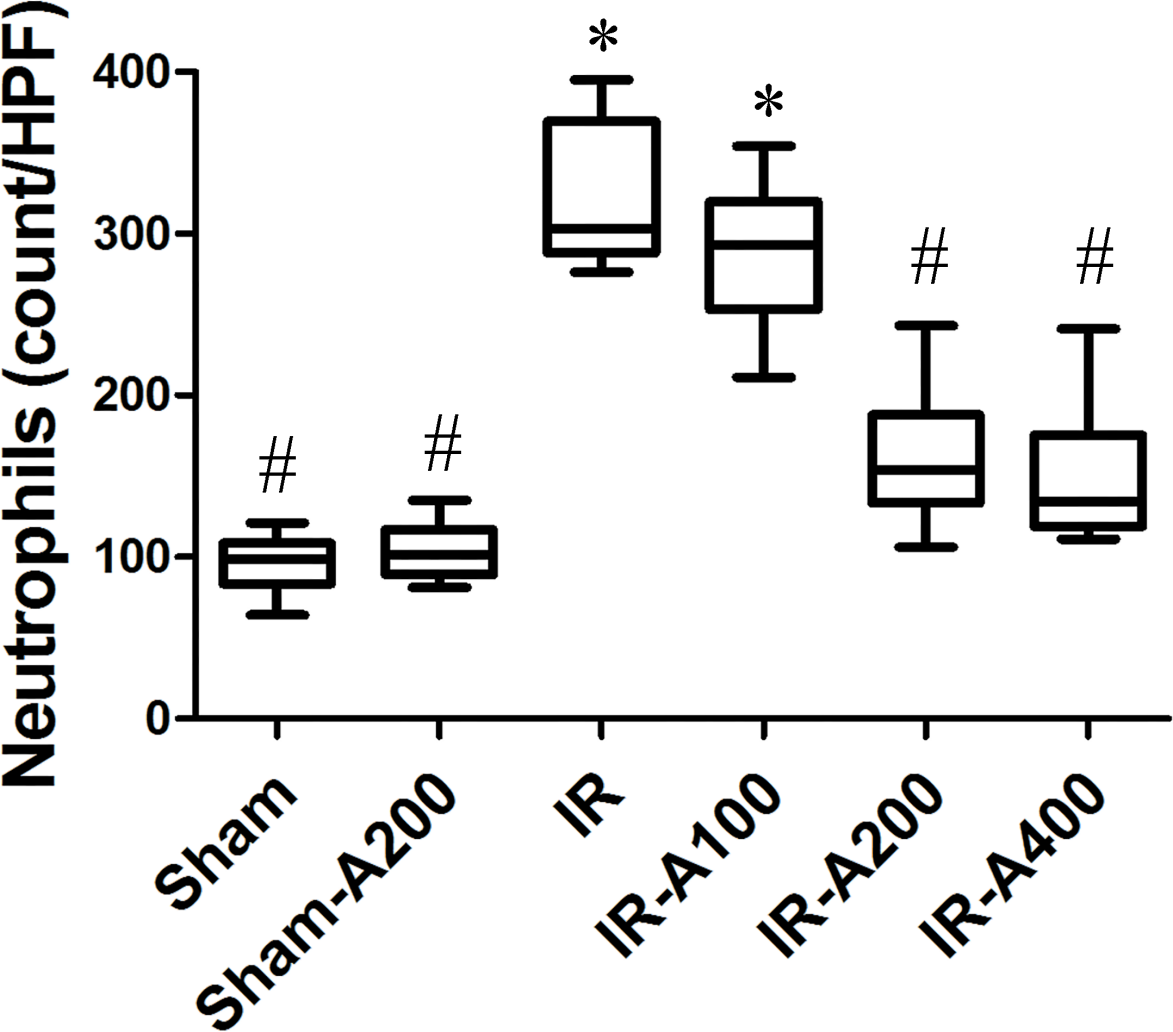
AZA attenuated neutrophilic sequestration in IR-induced ALI. Neutrophilic sequestration was markedly increased in the IR group (p<0.05 as compared with the sham group) and was significantly decreased by AZA 200 mg/kg BW and 400 mg/kg BW after IR (*p*<0.05 as compared between the IR and IR-AZA groups).There was a significant difference from the * Sham (*p*<0.05) and ^#^IR (*p*<0.05) groups. Sham group; Sham-A200, Sham + AZA 200mg/kg BW group; IR, ischemia-reperfusion group; IR-A100, IR + AZA 100mg/kg BW group; IR-A200, IR + AZA 200mg/kg BW group; IR-A400, IR + AZA 400mg/kg BW group.

### AZA attenuated alveolar protein leakage in IR-induced ALI (Fig 8)

Total protein concentration in BALF was significantly greater in IR (*p*<0.05 as compared with the sham group). Total protein concentration was significantly decreased by AZA 200 mg/kg BW and 400 mg/kg BW after IR (*p*<0.05 as compared between the IR and the IR with AZA groups).

**Fig 8:**
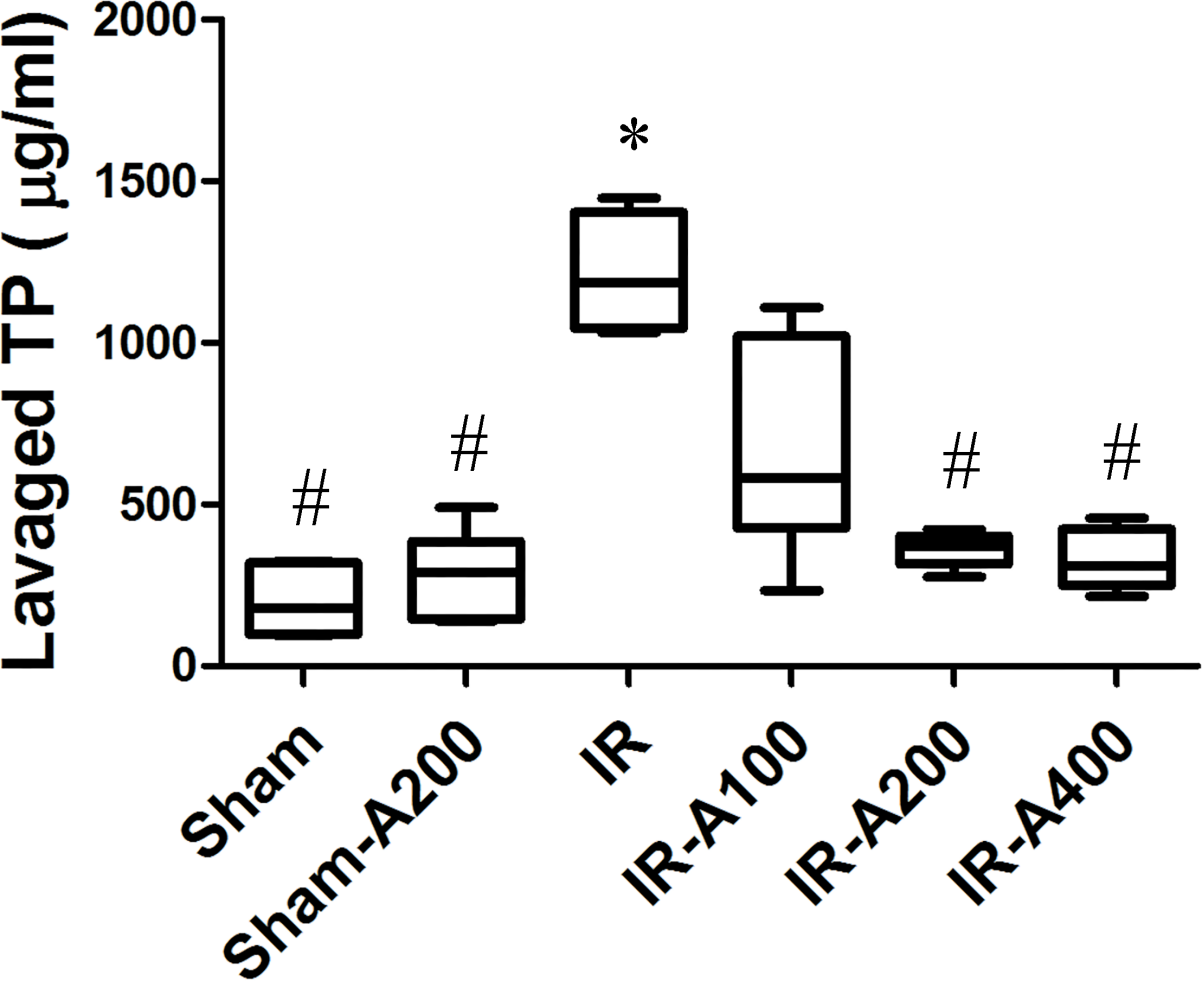
AZA attenuated alveolar protein leakage in IR-induced ALI. Total protein (TP) concentration in bronchoalveolar lavage fluid (BALF) was significantly increased in IR (*p*<0.05 as compared with the sham group) and was significantly decreased by AZA 200 mg/kg BW and 400 mg/kg BW after IR (*p*<0.05 as compared between the IR and IR-AZA groups). There was a significant difference from the * Sham (*p*<0.05) and ^#^IR (*p*<0.05) groups. Sham group; Sham-A200, Sham + AZA 200mg/kg BW group; IR, ischemia-reperfusion group; IR-A100, IR + AZA 100mg/kg BW group; IR-A200, IR + AZA 200mg/kg BW group; IR-A400, IR + AZA 400mg/kg BW group.

### AZA decreased IR-induced expression of pro-inflammatory cytokines (Fig 9)

Expression of pro-inflammatory cytokines (TNF-α, IL-1, IL-6 and IL-17) was significantly increased in the IR group (*p*<0.05 as compared with the sham group). Expression of these pro-inflammatory cytokines was significantly decreased by AZA 200 mg/kg BW and 400 mg/kg BW after IR (*p*<0.05 as compared between the IR and the IR with AZA groups).

**Fig 9:**
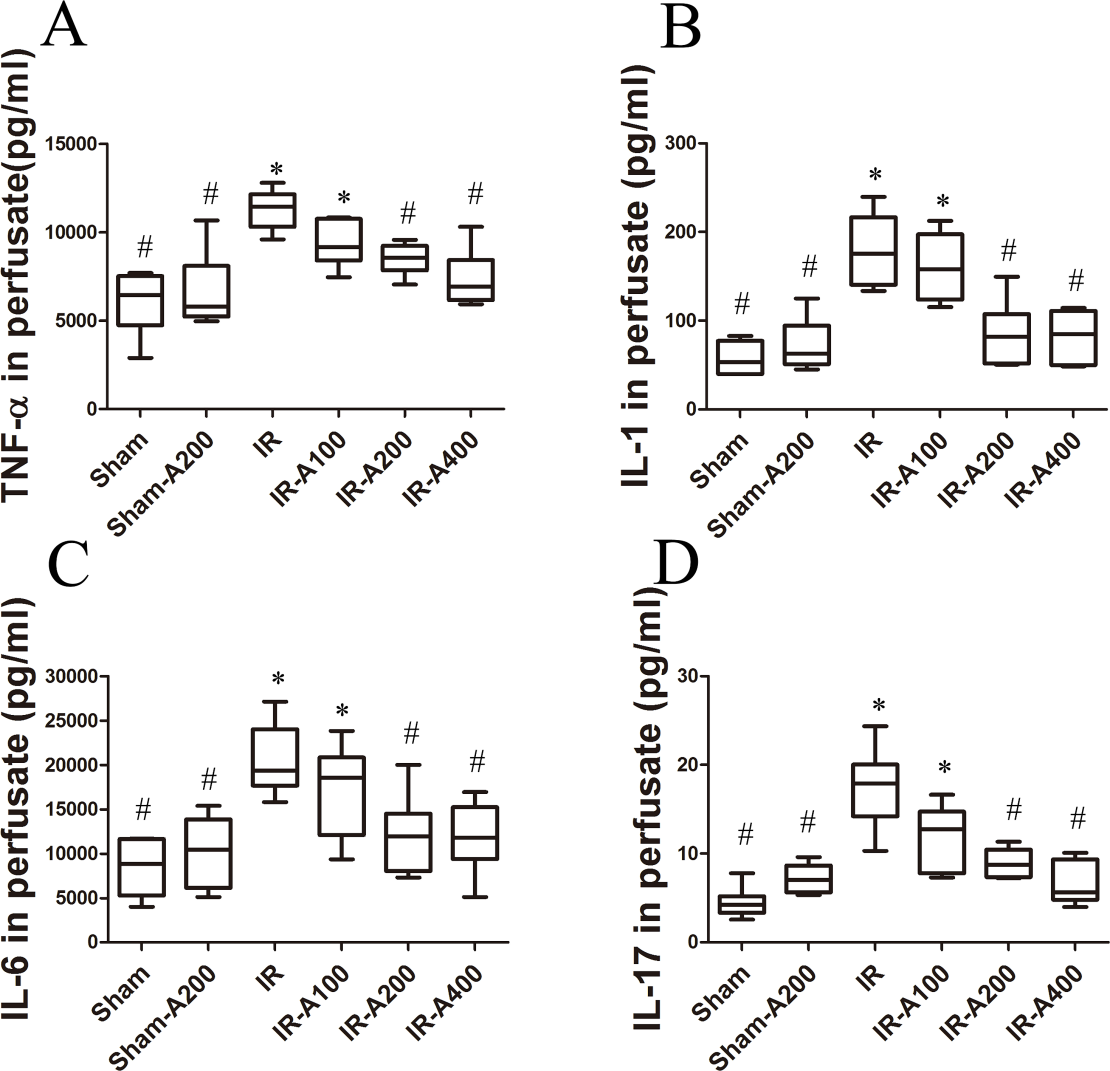
AZA decreased IR-induced expression of pro-inflammatory cytokines. Cytokines (TNF-α, IL-1, IL-6 and IL-17) in perfusate were significantly increased in the IR group (*p*<0.05 as compared with the sham group). The expression of these pro-inflammatory cytokines was significantly decreased by AZA 200 mg/kg BW and 400 mg/kg BW after IR (*p*<0.05 as compared between the IR and IR-AZA groups). There was a significant difference from the * Sham (*p*<0.05) and ^#^IR (*p*<0.05) groups. Sham group; Sham-A200, Sham + AZA 200mg/kg BW group; IR, ischemia-reperfusion group; IR-A100, IR + AZA 100mg/kg BW group; IR-A200, IR + AZA 200mg/kg BW group; IR-A400, IR + AZA 400mg/kg BW group.

### Time course of pro-inflammatory cytokines (Fig 10)

**Fig 10:**
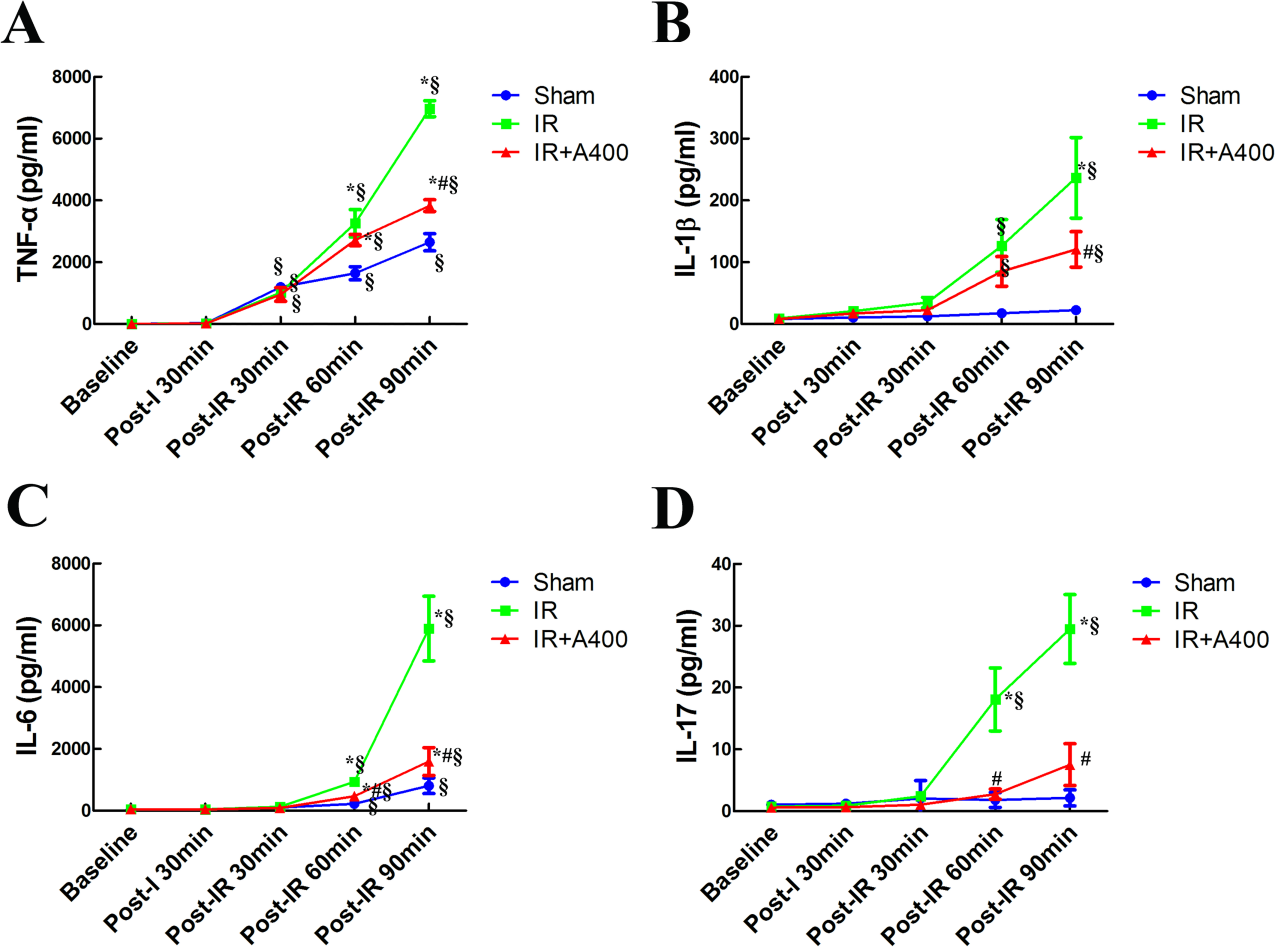
Time course of expression of pro-inflammatory cytokines. No significant differences in the expressions of TNF-α (A), IL-1 (B), IL-6 (C) and IL-17 (D) were observed at baseline in the three groups (*p*> 0.05 as compared amongthe sham, IR and IR with AZA groups at baseline). After post-reperfusion 90 minutes, these cytokines were significantly increased in the IR and IR-A400 groups (p<0.05 as compared with baseline between the IR and IR-A400 groups). The elevation of these cytokines were lowered by AZA 400 mg/kg BW (p<0.05 as compared between the IR and IR-A400 groups). In the sham group without insult, slight increase in cytokines remained after periods of experiments (p<0.05 as compared with baseline). The elevation of cytokines was significant less than that in the IR group at post-reperfusion 90minutes (*p*< 0.05 as compared with the IR group). There was a significant difference from the *Sham (*p*<0.05) and ^#^IR (*p*<0.05) groups and from ^§^baseline (*p*<0.05). Sham group; IR, ischemia-reperfusion group; IR-A400, IR + AZA 400mg/kg group.

Expression of pro-inflammatory cytokines (Fig 10) in these groups did not significantly differ between baseline and post-ischemia (*p*> 0.05 as compared between the sham, IR and IR with AZA groups at baseline and post-ischemia). At post-reperfusion 90 minutes, these cytokines levels were significantly increased in the IR and the IR-A400 groups (p<0.05 as compared with baseline). The elevation of these cytokines were attenuated by AZA 400 mg/kg BW (p<0.05 as compared between the IR and the IR A400 groups). Notably, the sham group also demonstrated a mild increase in expression of pro-inflammatory cytokines over the duration of the experiment (p<0.05 as compared with baseline). However, the elevation of cytokines were significant less than the IR group at post-reperfusion 90 minutes(*p*< 0.05 as compared with the IR group)

### mRNA expression of carbonic anhydrase II (Fig 11)

**Fig 11:**
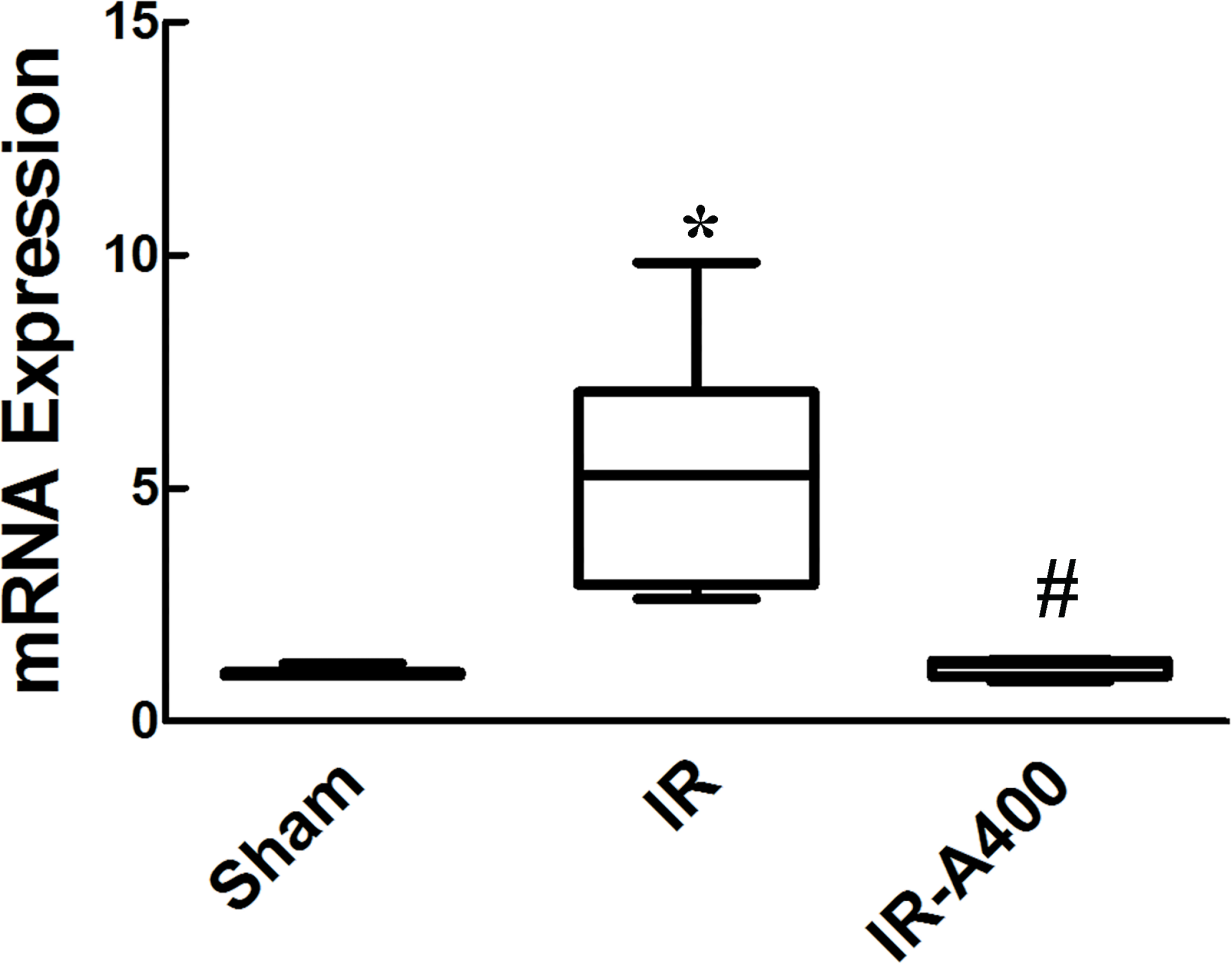
mRNA expression for carbonic anhydrase II. Higher expression of carbonic anhydrase II expression was found in the IR group(p<0.05 as compared between the sham and IR group). AZA decreased the expression of carbonic anhydrase II mRNA(p<0.05 as compared between the IR and IR-A400 group). There was a significant difference from the * Sham (*p*<0.05) and ^#^IR (*p*<0.05) groups. Sham group; IR, ischemia-reperfusion group; IR-A400, IR + AZA 400mg/kg group.

Low expression of carbonic anhydrase II mRNA (Fig 11) was detected in the sham group. However, in the IR group, higher expression of carbonic anhydrase II was found (p<0.05 as compared between the sham and the IR group). Administration of AZA 400mg/kg BW significantly decreased the expression of carbonic anhydrase II mRNA (p<0.05 as compared between the IR and the IR-A400 group).

### Immunofluorescent staining of carbonic anhydrase in lung tissues (Fig 12)

**Fig 12:**
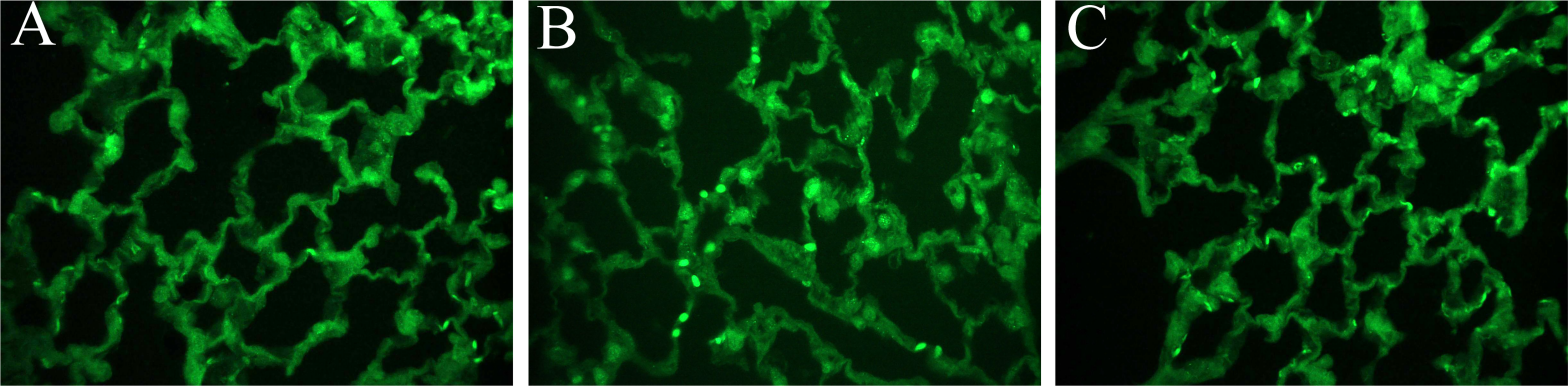
Immunofluorescent staining of carbonic anhydrase in lung tissues. Immunofluorescent staining of carbonic anhydrase II in lung tissues of the sham (A), IR (B) and IR-A400 (C). Enhanced staining (bright green dots) of carbonic anhyrase II was higher in the IR group than that in the sham group. AZA attenuated the enhanced expression of carbonic anhydrase II in lung tissues.

Representative immunofluorescent staining of carbonic anhydrase II in lung tissues of the sham (Fig 12A), IR (Fig 12B) and IR-A400 (Fig 12C) groups are shown. Enhanced staining (bright green dots) of carbonic anhyrase II was higher in the IR group than that in the sham group. AZA attenuated the enhanced expression of carbonic anhydrase II in lung tissues.

### Carbonic anhydrase activity in lung tissues (Fig 13)

**Fig 13:**
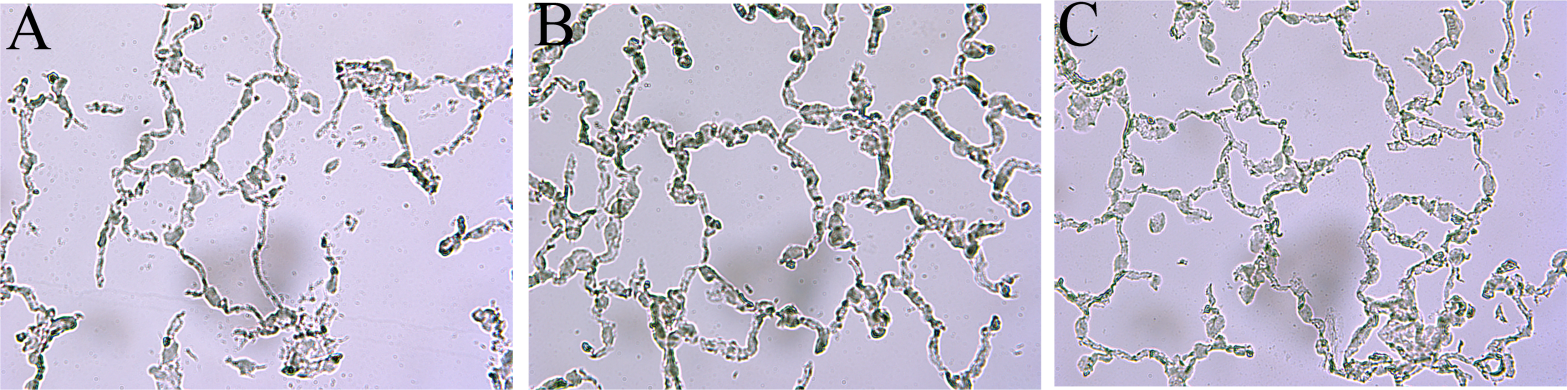
Hansson's staining of carbonic anhydrase activity in lung tissues. The IR group showed higher carbonic anhydrase activity than that in the sham group. AZA inhibited carbonic anhydrase activity in lung tissues after IR.

Carbonic anhydrase activity in lung tissues of the sham (Fig 13A), IR (Fig 13B) and IR-A400 (Fig 13C) groups are shown. The IR group showed higher carbonic anhydrase activity than that in the sham group. AZA inhibited carbonic anhydrase activity in lung tissues.

### Expression of membranous Na,K-ATPase α1 in lung tissues

**Fig 14:**
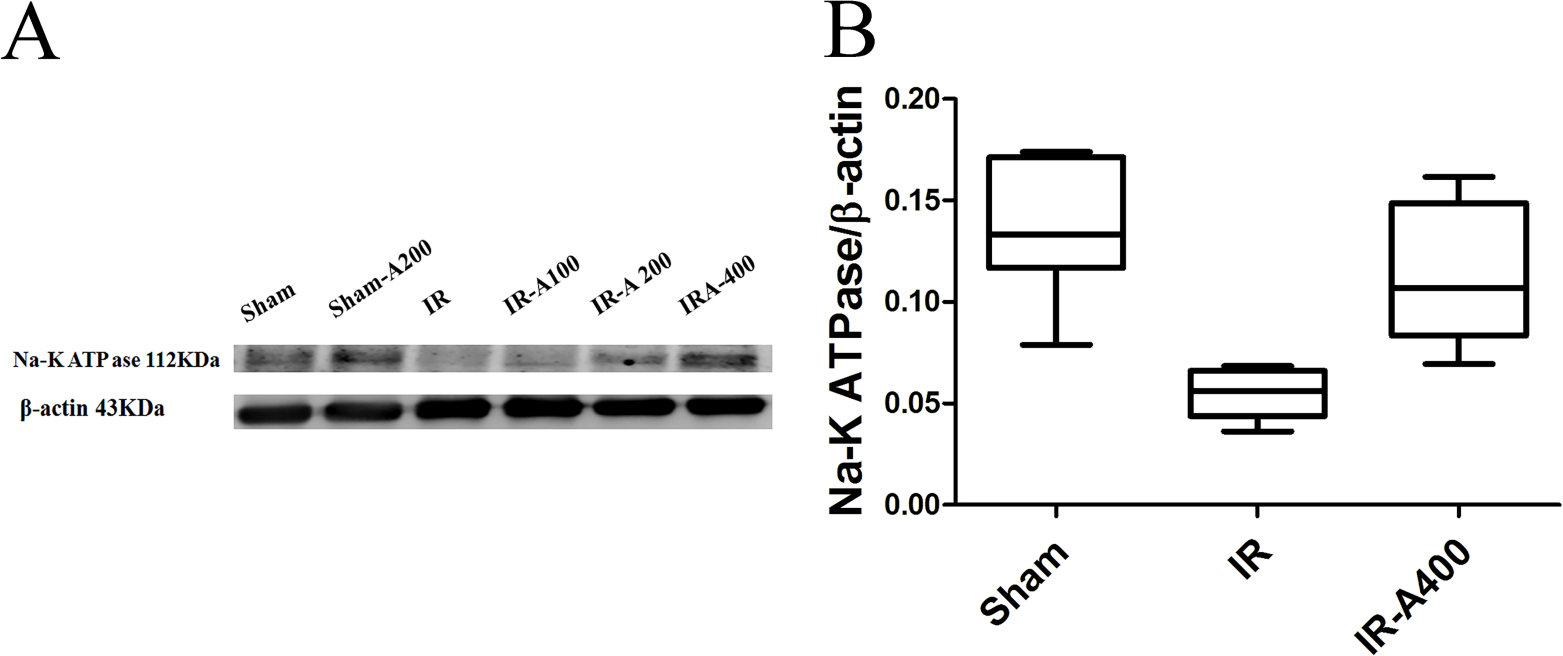
Membranous Na-K-ATPase α1 in lung tissues. Resperentative (A) and quantitative analyses (B) showed that Na-K-ATPase α1 expression was higher in the sham group than that in the IR group (p<0.05 as compared between the sham and IR group). IR-A400 group restored expression of Na-K-ATPase α1 (p<0.05 as compared between the IR and IR-A400 group). There was a significant difference from the * Sham (*p*<0.05) and ^#^IR (*p*<0.05) groups. Sham group; IR, ischemia-reperfusion group; IR-A400, IR + AZA 400mg/kg group.

Resperentative (Fig 14A) and quantitative analyses (Fig 14B) of Na-K-ATPase α1 of the sham, IR and IR-AZA 400 groups are shown in Fig 13. Expression of Na-K-ATPase α1was higher in the sham group than in the IR group. However, administration of AZA restored expression of Na-K-ATPase α1 to baseline levels.

## Discussion

The current study revealed that IR-induced ALI caused increased pulmonary micro-vascular permeability, pulmonary edema, pulmonary hypertension, neutrophilic sequestration, and pro-inflammatory cytokines. IR also presented with an increase in expression and activity of carbonic anhydrase, higher pCO_2_ and a decrease expression of Na,K-ATPase. AZA inhibited expression and activity of carbonic anhydrase, lowered pCO_2_ level and restored expression of Na,K-ATPase. Consequently, treatment with AZA significantly attenuated IR-induced lung injury with decreased pulmonary micro-vascular permeability, lung inflammation, pulmonary edema, and pulmonary hypertension.

To our knowledge, this is the first study of AZA treatment for IR-induced ALI. There were a few studies on AZA in IR in other organs. Bejaoui et al. found that AZA protected liver grafts against IR injury[17]; Lin and colleagues suggested that AZA decreased infarct size in cardiac IR[18]; An et al. demonstrated that AZA exerted a reno-protective role against I/R-induced acute renal injury[19]. In another study, researchers investigated the role of AZA in intracerebral hemorrhage(ICH)-induced brain injury in rats[20]. They found AZA was able to reduce ICH-induced brain edema and neuronal deaths. Based on these studies, it is reasonable to assert that AZA is capable of producing protective effects against IR injuries in many organs. In the present study, we further addressed that AZA was beneficial in treating IR-induced lung injury as well.

The pathogenesis of IR-induced ALI involves several biochemical, cellular, and molecular alterations[21]. Depletion of cellular energy and the accumulation of toxic oxygen metabolites contribute to ischemic lung injury[21]. Tissue damage is exacerbated at the moment of reperfusion, and is considered more harmful than ischemia alone[21]. Reperfusion related injury is directly related to the formation of reactive oxygen species, endothelial cell injury, increased vascular permeability, and activation of neutrophils, cytokines, and complement system[21]. The present study demonstrated the protective effects of AZA via regulation of CO_2_ levels and inflammation after IR.

During ischemia, O_2_ delivery is stopped, and the cells convert to anaerobic metabolism, which produces lactic acid, protons and other toxic byproducts. Protons buffered by HCO_3_^-^ form CO_2_ in the tissues[22]. In the present study, pCO_2_ was increased after ischemia, indicating that increased CO_2_ production happens during anaerobic metabolism.

In the current study, increased activity and expression of carbonic anhydrase were noted after IR. Carbonic anhydrase accelerates the conversion of H_2_CO_3_^-^ to CO_2_ in the lungs[23]. Therefore, higher CO_2_ level was found in the IR group. Higher CO_2_ level contributed to further deterioration of alveolar epithelial function and impairment of alveolar fluid absorption[11]. However, administration of AZA in perfusate inhibited carbonic anhydrase activity[24], slowed the conversion of HCO_3_^-^ to CO_2_ and decreasing CO_2_ levels[8],[24]. Therefore, AZA has protective effects for lung injury by decreasing CO_2_ level in lungs.

Intracellular CO_2_ affects the alveolar epithelial function[11]. Active Na^+^ transport affects edema clearance with water following Na^+^ gradient via apically located sodium channels and basolaterally-located Na,K-ATPase[25]. Epithelial Na,K-ATPase is a major contributor to alveolar fluid reabsorption[25]. Briva et al. found that when alveolar epithelial cells were exposed to high CO_2_, the Na,K-ATPase activity is decreased[11]. A decrease in Na,K-ATPase activity resulted in the inhibition of Na^+^ transport, with followed by inhibition of alveolar fluid reabsorption[26]. High CO_2_ level triggered endocytosis and inhibition of Na,K-ATPase activity in alveolar epithelial cells[11], which suggested that elevated CO_2_ level decreased alveolar fluid reabsorption[11]. Effros et al. reported similar findings[24] when isolated fluid–filled rat lungs were perfused with a solution that contained 40 or 60 mmHg CO_2_,–alveolar fluid reabsorption was reduced by 50% in hypercapnic conditions [24]. Vadasz et al also foundthat CO_2_ accumulation resulted in deleterious effects in the lungs[10]. They suggested that elevated CO_2_ level caused damage to the epithelial and endothelial barrierand compromised host defense [10].

We found that AZA influenced the expression of pro-inflammatory cytokines. Pulmonary IR injury involves neutrophils, T cells and pro-inflammatory cytokines[21]. TNF-α is a potent cytokine released early in response to IR[21] and stimulates neutrophils to release inflammatory mediators leading to further damage[21]. Administration of AZA significantly attenuated lung inflammation, neutrophils infiltration and expressions of pro-inflammatory cytokines. These findings suggested that AZA is capable to attenuate IR-induced ALI mediated by an anti-inflammatory mechanism.

Prior studies have addressed the anti-inflammatory effects of AZA[17, 27]. Wang et al. found that AZA could decrease pro-inflammatory cytokines, such as monocyte chemoattractant protein-1, IL-1β, TNF-α and Interferon gamma in rat lung injury of acute mountain sickness[27]. They suggested that AZA protected lung tissue from injury by reducing expression of pro-inflammatory cytokines[27]. Elevated mitogen activated protein kinases (MAPKs) are found in lung injury[28]. It has been reported that treatment with AZA may cause decreased expression of MAPKs[17].

IL-17 is expressed by activated T cells[29]. Lymphocytes also play an important role in IR-induced ALI. In the model of a rat lung transplant[21], it was observed that CD^4+^ T lymphocytes infiltrated the graft after reperfusion[21]. Sharma et al. suggested that CD^4+^ invariant natural killer T cells and IL-17 dependent mechanism is important in IR lung injury[29]. IL-17 is a potent mediator of activation, recruitment and infiltration of neutrophils[29]. In our current study, expression of IL-17 was increased after IR induction and administration of AZA decreased the expression of IL-17. This implies that AZA may also inhibit the pathways of CD4^+^ T cells and the IL-17 dependent mechanism.

In this study, we noted that the expression of pro-inflammatory cytokines was increased in the sham group. This indicated that the procedures of lung isolation with mechanical ventilation resulted in the elevation of pro-inflammatory cytokines. However, this degree of elevation was minimal as compared with the IR group. Veldhuizen et al. showed a similar result[30], where increased expression of pro-inflammatory cytokinesoccurred when the lungs were isolated and ventilated with a tidal volume 7ml/kg[30]. They suggested that this injury was partly mediated by the surfactant system and the releases of pro-inflammatory cytokines following mechanical ventilation[30].

### Clinical implications

Lungs are vulnerable and IR-induced ALI has beenimplicated in many clinical conditions such as lung transplantation, pulmonary thrombo-embolectomy, and cardio-pulmonary by-pass surgery[3–5]. The mortality of IR-induced ALI remains high despite modern medical managements. Understanding the relationship between carbonic anhydrase and IR-induced ALI may facilitate development of novel therapeutic interventions.

AZA, a carbonic anhydrase inhibitor, is an effective drug for the prevention of high-altitude cerebral edema and management of idiopathic intracranial hypertension and glaucoma[20]. In the present study, we showed that AZA can attenuate IR-induced ALI. This study may support the use of AZA in the treatment of IR-induced ALI in the clinical setting.

### Limitations of study

The present study revealed a possible, AZA-based therapy for IR-induced ALI. However, there are still limitations in this study. First, it was performed on animal species with isolated lung preparations. For the future, studies involving human beings in clinical settings are warranted. Second, cellular effects of AZA and intracellular CO_2_ levels are important, but measurement of intracellular CO_2_ levels is not possible in the isolated lung model employed in the present study. Despite this limitation, AZA was still proven to be a beneficial drug for IR-induced lung injury. Besides, CO_2_ levels in perfusate was measured, and it was noted that, after IR, pCO_2_ was lower in the IR-A400 group than in the IR group. In the IR group, higher carbonic anhydrase enhances conversion of HCO_3_^-^ to CO_2_. AZA inhibited carbonic anhydrase and slowed down the conversion of HCO_3_^-^ into CO_2_. This result still supported higher CO_2_ level in the IR group and higher CO_2_ impairs alveolar epithelial function. Third, it was interesting to find an increased expression and activity of carbonic anhydrase after IR. The mechanism underlying this increase of carbonic anhydrase was unknown. Future studies seeking to confirm this mechanism are necessary.

## Conclusions

The carbonic anhydrase inhibitor, AZA has protective effects on subjects ofIR-induced lung injury. AZA can decrease expression and activity of carbonic anhydrase, lower pCO_2_ level, restore Na,K-ATPase and then attenuate pulmonary microvascular hyper-permeability, pulmonary edema, pulmonary hypertension, and neutrophilic sequestration in the lungs after IR. AZA also decreases IR-induced expression of pro-inflammatory cytokines. This investigation provides support for the use of carbonic anhydrase inhibitors in the clinical treatment of IR-induced ALI. Further investigations are still needed to verify the responses of carbonic anhydrase inhibitors in ALI in human beings.

## Supporting information

S1 File Data of perfusate blood gas. The mean pCO_2_, pH, and HCO_3_^-^ values at baseline were similar across all groups. In the IR and IR-A400 groups, pCO_2_ levels increased after ischemia (*p*<0.05 as compared with baseline). Following reperfusion and ventilation, pCO_2_ levels in the IR-A400 group were lower than that in the IR group (p<0.05 as compared between the IR and IR-A400 groups). The pH was stationary in the sham and IR-A400 groups. However, pH was decreased in the IR group (p<0.05 as compared with baseline; p<0.05 as compared between the IR and sham, and IR-A400 groups). HCO_3_^-^ had similar changes as pH during ischemia and reperfusion.
(XLS)

S2 File Data of pulmonary edema. Lung weight/body weight (LW/BW) and wet/dry weight (W/D) ratios were significantly higher in the IR than sham groups (*p*<0.05 as compared between the IR and sham). The IR-A200 and IR-A400 groups had significantly lower LW/BW and W/D than the IR group (*p*<0.05 as compared between the IR and IR with AZA groups).
(XLSX)

S3 File Data of pulmonary microvascular permeability. The Kf1 at baseline was similar among all groups (*p*>0.05 as compared among the sham, IR and IR with AZA groups). The Kf2 in the IR group was significantly higher than that in the sham group (*p*<0.05 as compared between the IR and sham groups). AZA significantly attenuated IR-induced microvascular hyper-permeability (*p*<0.05 as compared with the IR group). Changes of micro-vascular permeability were significantly higher in the IR group (*p*<0.05 as compared with the sham group) and were attenuated by administration of AZA (*p*<0.05 as compared between the IR and IR with AZA groups).
(XLSX)

S4 File Data of pulmonary arterial pressure. The PAP at baseline was similar among these groups (*p*>0.05 as compared among the sham, IR and IR with AZA groups). After IR, the PAP levels were significantly increased in the IR group (*p*<0.05 as compared with the sham group). AZA significantly decrease the PAP levels after IR (*p*<0.05 as compared between the IR and IR with AZA groups). Changes of PAP levels were significantly increased in the IR group (*p*<0.05 as compared with the sham group) and were attenuated by AZA (*p*<0.05 as compared between the IR and IR with AZA groups).
(XLSX)

S5 File Data of neutrophil count. Neutrophilic sequestration was markedly increased in the IR group (p<0.05 as compared with the sham group) and was significantly decreased by AZA after IR (*p*<0.05 as compared between the IR and IR-AZA groups).
(XLSX)

S6 File Data of total protein in BALF. Total protein concentration in bronchoalveolar lavage fluid was significantly increased in IR (*p*<0.05 as compared with the sham group) and was significantly decreased by AZA after IR (*p*<0.05 as compared between the IR and IR-AZA groups).
(XLSX)

S7 File Data of pro-inflammatory cytokines in perfusate. Cytokines (TNF-α, IL-1, IL-6 and IL-17) in perfusate were significantly increased in the IR group (*p*<0.05 as compared with the sham group). The expression of these pro-inflammatory cytokines was significantly decreased by AZA after IR (*p*<0.05 as compared between the IR and IR-AZA groups).
(XLSX)

S8 File Data of time course of expression of pro-inflammatory cytokines. No significant differences in the expressions of TNF-α, IL-1, IL-6 and IL-17 were observed at baseline (*p*> 0.05 as compared amongthe sham, IR and IR with AZA groups at baseline). After post-reperfusion 90 minutes, these cytokines were significantly increased in the IR and IR-A400 groups (p<0.05 as compared with baseline between the IR and IR-A400 groups). The elevation of these cytokines were lowered by AZA (p<0.05 as compared between the IR and IR-A400 groups).
(XLSX)

S9 File Data of mRNA expression for carbonic anhydrase II. Higher expression of carbonic anhydrase II expression was found in the IR group (p<0.05 as compared between the sham and IR group). AZA decreased the expression of carbonic anhydrase II mRNA (p<0.05 as compared between the IR and IR-A400 group).
(XLSX)

S10 File Data of membranous Na-K-ATPase α1 in lung tissues. Na-K-ATPase α1 expression was higher in the sham group than that in the IR group (p<0.05 as compared between the sham and IR group). IR-A400 group restored expression of Na-K-ATPase α1 (p<0.05 as compared between the IR and IR-A400 group).
(XLSX)
